# Circulating Cell-Free DNA Levels Could Predict Oncological Outcomes of Patients Undergoing Esophagectomy for Esophageal Squamous Cell Carcinoma

**DOI:** 10.3390/ijms17122131

**Published:** 2016-12-17

**Authors:** Chih-Cheng Hsieh, Han-Shui Hsu, Shih-Ching Chang, Yann-Jang Chen

**Affiliations:** 1 Institute of Clinical Medicine, School of Medicine, National Yang-Ming University, Taipei 11221, Taiwan; cchsieh2@vghtpe.gov.tw; 2 Division of Thoracic Surgery, Department of Surgery, Taipei Veterans General Hospital, Taipei 11217, Taiwan; hsuhs@vghtpe.gov.tw; 3 Department of Surgery, School of Medicine, National Yang-Ming University, Taipei 11221, Taiwan; changsc@vghtpe.gov.tw; 4 Institute of Emergency and Critical Care Medicine, School of Medicine, National Yang-Ming University, Taipei 11221, Taiwan; 5 Division of Colon and Rectal Surgery, Department of Surgery, Taipei Veterans General Hospital, Taipei 11217, Taiwan; 6 Department of Life Sciences and Institute of Genome Sciences, National Yang-Ming University, Taipei 11221, Taiwan; 7 Department of Pediatrics, Renai Branch, Taipei City Hospital, Taipei 10629, Taiwan

**Keywords:** circulating cell-free DNA, esophageal squamous cell carcinoma, survival

## Abstract

Circulating cell-free DNA (cfDNA) is a potential biomarker for cancer progression but its role is unclear in patients with esophageal squamous cell carcinoma (ESCC) after esophagectomy. We investigated relationships between plasma cfDNA levels and clinicopathological parameters in ESCC patients. Eighty-one ESCC patients who received esophagectomy were enrolled. Plasma samples from these patients and 95 normal controls were collected. DNA copy numbers were measured by real-time quantitative PCR. Subjects were divided into two groups by cfDNA level. Clinicopathological data were collected retrospectively and relationships between cfDNA levels and clinical parameters were evaluated. The cfDNA level in normal controls ranged from 0–4157 copies/mL. The cfDNA level of 96.3% ESCC patients was higher than the cutoff value (2447.26 copies/mL) with a specificity of 94.1%. The mean cfDNA concentration was 5918 copies/mL in lower and 53,311 copies/mL in higher cfDNA groups. No correlations were found between clinicopathological factors and cfDNA levels except for lymphovascular invasion. Higher cfDNA levels were associated with tumor relapse (*p* = 0.018). Five-year disease-free survival (DFS) and overall survival (OS) rates were 34.7% and 33.8%, respectively. Patients with higher cfDNA levels had poorer DFS (*p* = 0.013). Patients with higher cfDNA levels had poorer OS, but not significantly (*p* = 0.164). Circulating cfDNA could be a biomarker for tumor relapse of ESCC with high sensitivity and specificity. Higher cfDNA levels were associated with tumor relapse and shorter DFS after esophagectomy in ESCC patients.

## 1. Introduction

The incidence of esophageal cancer is increasing all over the world. The main treatment methods for esophageal cancer include surgical resection, chemotherapy, and radiotherapy. However, although the treatment for esophageal cancer has advanced greatly in recent decades, the treatment outcomes are still poor, and the five-year survival rate is less than 15% [1,2,3,4]. Poor outcomes are mainly due to delayed diagnosis as a result of late presentation of symptoms or structural changes. In order to achieve a higher cure rate in esophageal cancer, early detection of the primary or recurrent disease is essential [5].

Specific clinical symptoms and signs are not usually helpful in making an early diagnosis and results of most diagnostic studies are not reliable. Widespread screening is usually not possible and may only result in the incidental discovery of small tumors in the esophageal cancer population [5,6]. In clinical practice, the pathological stage of cancerous disease predicts clinical outcome more profoundly than any other markers available currently for individual patients who have received surgical treatment for esophageal cancer [5]. However, the data of the pathological stage is based on post-operative examination of resected specimens. Pre-treatment evaluation or repeat measurements represent a significant diagnostic challenge.

A noninvasive method for early detection of esophageal cancer and advanced indications for additional therapy could represent important clinical advances in patient management [5]. In 1948, Mandel and colleagues reported the existence of circulating extracellular nucleic acids in human blood [7]. In recent decades, many studies have found that circulating cell-free DNA (cfDNA) was present in higher levels among patients with certain malignant diseases as compared to levels in healthy individuals [8,9,10,11,12]. Although many studies have focused on the clinical relationships between cfDNA and different types of solid tumors, very few have investigated esophageal cancers. Therefore, the aim of the study was to analyze the relationships between the levels of plasma cfDNA and the clinicopathological parameters in patients with esophageal squamous cell carcinoma (ESCC).

## 2. Results

### 2.1. Clinical and Pathological Data

The mean age of the 81 patients with ESCC was 60.4 ± 11.5 years, ranging from 38 to 84 years. Seventy (86.4%) patients were male and 11 were female. Fifty-eight (71.6%) patients were smokers and 39 patients smoked more than 20 package-years. Twenty-six (32.1%) patients had never consumed alcohol and 17 patients engaged in social drinking or had quit drinking for more than five years. Fifteen (18.5%) patients had a history of chewing betel nuts. Eleven (13.6%) tumors were found at the cervical or upper portion of the thoracic esophagus, 35 (43.2%) at the middle portion and the other 35 at the lower portion of the esophagogastric junction.

All patients received an esophagectomy with cervical anastomosis, 44 (54.3%) through open thoracotomy, 34 (42.0%) with thoracoscopic assistance, and the transhiatal approach was used in three patients to perform the esophagectomy. A gastric tube was used for reconstruction in all patients, 40 (49.4%) through the posterior mediastinal route and 41 (50.6%) through the retrosternal route. One (1.2%) surgical mortality occurred due to postoperative myocardial infarction.

Based on the AJCC classification for esophageal cancer, seven (8.6%) patients had pathological T1 lesions, 17 (21.0%) had T2, 52 (64.2%) had T3, and five (6.2%) had T4. The average number of lymph nodes removed was 25.9 (range of two to 67). For pathological N status, 31 (38.3%) patients had N0, 27 (33.3%) had N1, 14 (17.3%) had N2, and nine (11.1%) had N3 status. For pathological stages, five (6.2%) patients had stage I, 32 (39.5%) had stage II, and 44 (54.3%) had stage III disease. The pathological stages I–II correlated with the T1–2 status (*p* < 0.001) and N0 status (*p* < 0.001).

The maximum tumor size of the surgical specimens was defined by the tumor length. In the present study, the median tumor length was 4.0 cm (range of 1.3–8.3 cm). Tumor length was significantly smaller in the pathological T1–2 status (*p* = 0.003) but did not correlate with stages I–II (*p* = 0.068). Forty-six (56.8%) patients received postoperative adjuvant treatment for locally advanced disease or lymph node metastasis, including 34 patients with concurrent chemoradiotherapy, eight with chemotherapy only and four with radiotherapy alone. The adjuvant treatment correlated with T3–4 (*p* = 0.002), positive nodal status (*p* = 0.001) and stage III disease (*p* < 0.001). 

### 2.2. Plasma cfDNA Concentration

**Figure 1:**
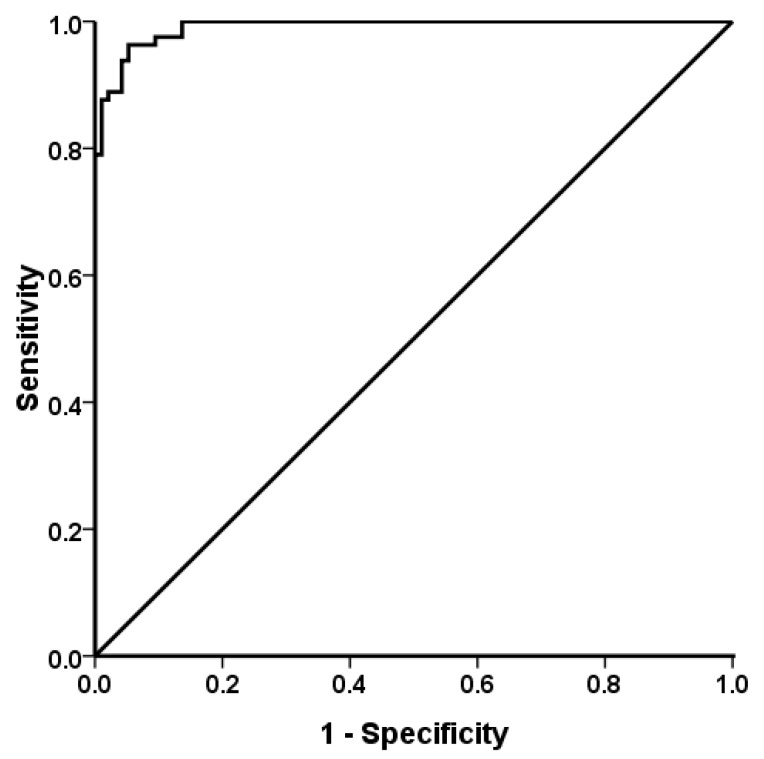
Receiver operating characteristic curve of plasma circulating cell-free DNA in 81 patients with esophageal squamous cell carcinoma and 95 normal controls. The sensitivity was 96.3% and the specificity was 94.1%; the area under the curve was 0.991 (95% confidence interval 0.982–0.999).

The levels of cfDNA in 95 normal controls (66 males and 29 females, mean age 54.2 ± 15.5 years) ranged from 0–4157 copies/mL (mean 613 ± 888; median 168 copies/mL). The levels of cfDNA varied widely between patients, ranging from 1687 to 161,170 copies/mL (mean 29,907 ± 35,755; median 14,090 copies/mL). There was a significant difference between the normal controls and patients (*p* < 0.001). According to the receiver operating characteristic (ROC) curve, the cutoff value was 2447.26 copies/mL for the diagnosis of esophageal cancer. The sensitivity was 96.3% and the specificity was 94.1%; the area under the ROC curve was 0.991 (Figure 1). 

All patients were divided into two groups according to the median level of cfDNA (lower and higher levels); one group included 40 patients with lower cfDNA levels (mean cfDNA: 5918 ± 3488 copies/mL) and the other group included 41 patients with higher cfDNA levels (mean cfDNA: 53,311 ± 37,524 copies/mL). As shown in Table 1, no significant correlations were observed between most clinicopathological parameters and lower and higher cfDNA levels. Only lymphovascular invasion correlated positively with higher cfDNA levels (*p* = 0.033).

### 2.3. Survival Data

The median follow-up time after surgery was 30.0 months, ranging from 3.7 to 115.9 months except for one surgical mortality case. Prior to data analysis, 49 (60.5%) patients had tumor relapse, including five local recurrences and 44 distal or multiple metastases. Among these patients, 30 (61.2%) patients with tumor relapse had higher cfDNA levels (*p* = 0.018). The median disease-free survival (DFS) of patients with tumor relapse was 11.5 months, ranging from 1.6 to 44.9 months. Six patients were still alive after treatment for disease relapse and the median follow-up time for these six patients was 40.9 months, ranging from 22.2 to 86.2 months. The three-year and five-year DFS rates were 36.5% and 34.7%, respectively.

**Figure 2:**
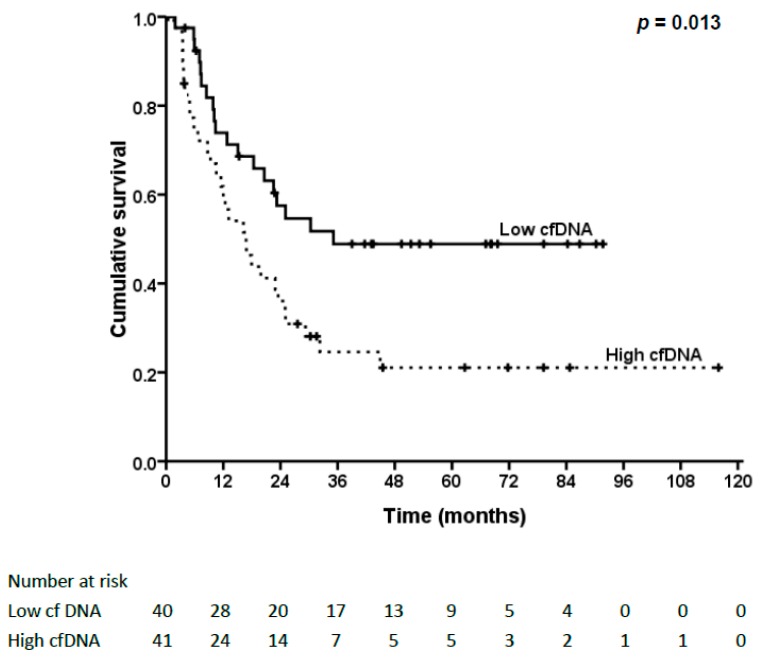
Kaplan–Meier survival curve and log-rank test of different cfDNA levels in disease-free survival (DFS) of esophageal squamous cell carcinoma (ESCC) patients. Patients with lower cfDNA levels had significantly better DFS (*p* = 0.013).

The survival analysis for DFS is shown in Table 2. Patients with N0 status, stage I–II or absent lymphovascular invasion had better DFS. In addition, patients with lower cfDNA levels had significantly better DFS (48.9% vs. 21.2%, *p* = 0.013, Figure 2). Nevertheless, no significant correlations were found between cfDNA levels and N status or stage.

**Figure 3:**
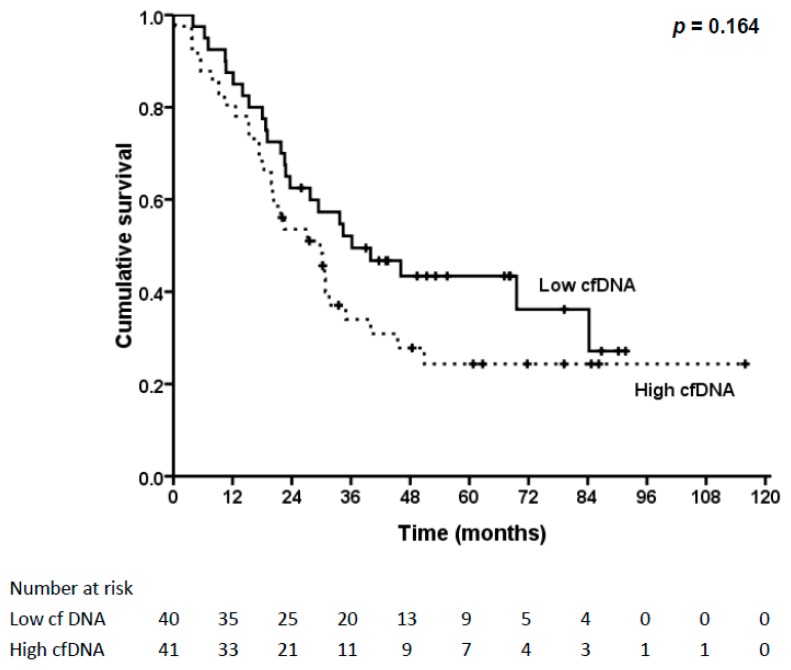
Kaplan–Meier survival curve and log-rank test of different cfDNA levels in overall survival (OS) of ESCC patients. Patients with lower cfDNA levels had better OS, but not significantly (*p* = 0.164).

A total of 43 (53.1%) patients died of the disease within the follow-up period, all within five years. Another 10 patients died of non-esophageal cancer diseases, including eight patients with pneumonia. The three-year and five-year overall survival (OS) rates were 43.1% and 33.8%, respectively. The survival analysis for OS is shown in Table 2. Patients with N0 status and stage I–II had better OS. Patients with lower cfDNA levels also had better OS, but not significantly (43.4% vs. 24.3%, *p* = 0.164, Figure 3).

## 3. Discussion

Even with advances in treatment, survival in patients with esophageal cancer is still poor. Early detection of tumors is essential to resolve this problem. Because the specific symptoms/signs of ESCC are not helpful for early tumor detection and diagnostic tools are not able to be applied in population screening [6], useful non-invasive biomarkers in blood samples are considered to be valuable and convenient for the early detection and subsequent management of cancers [11,12]. However, due to their low sensitivity and insufficient specificity, markers such as squamous cell carcinoma antigen (SCC) and cytokeratin 19 fragment (CYFRA) have little practical use in the early detection of ESCC [13,14]. Tracing the discovery history of biomarkers, the increased concentration of certain biochemical molecules in the blood, such as circulating nucleic acid, has been associated with different malignancies; as such, cfDNA has been suggested to be tumor-derived and its level may be associated with the disease burden and progression [10,15,16]. Thus, circulating cfDNA could be a potentially useful marker in esophageal cancer.

From simple qPCR methods to complex BEAMing technologies and deep next-generation sequencing, the methods used for cfDNA quantification have changed and the sensitivity and specificity of analysis have improved over time [15,17,18]. Some reviews discussed the problems of cfDNA measurements, such as the choice of plasma or serum, sample processing and storage, and DNA isolation and quantification [19,20]. However, there were no references discussing whether the blood sample was drawn preoperatively or postoperatively. Even the mechanism of the occurrence of cfDNA in blood was not fully understood, and several processes of cfDNA were discussed [21]. How the surgery influences cfDNA release into blood remains unknown. In this study, we collected the blood samples just after the operation, and it might represent the real amount of cfDNA influencing survival.

Many studies have suggested the cfDNA level is increased in different cancer patients compared to healthy individuals [8,9,10,11,12]. However, a recent meta-analysis demonstrated inconsistent results [19]. It is important to establish a normal reference for further study of the potential role of cfDNA as a useful marker and its utility in the early detection of recurrence [18]. However, the uses of different methods for cfDNA quantification, in addition to inconsistent reporting, have influenced a valid comparison of the results from different studies [18]. Furthermore, Ocaña et al. showed the cutoff values of cfDNA in different cancers in a meta-analysis of 39 studies, but no ESCC study was included [22]. Additionally, there were several studies that demonstrated that the concentration of cfDNA in esophageal cancer was higher than in normal individuals [23,24,25]. Tomochika and his coworkers set the cutoff value using the highest cfDNA value in patients without ESCC [23]. In this study, we showed that the levels of cfDNA in ESCC patients were higher than those in normal controls and the cutoff value of circulating cfDNA in ESCC patients was established with high sensitivity and specificity according to the ROC curve. Considering the potential biases affecting the results of the ROC curve, sample size was the primary factor that influenced the sensitivity and specificity in this retrospective study; further study with a large sample size is needed. 

In the present study, cfDNA was evaluated as a possible factor in the survival of patients with ESCC. We divided all postoperative ESCC patients into two groups according to the median level of cfDNA, including those with lower and higher cfDNA levels. No associations were found between levels of cfDNA and most clinicopathological parameters, including gender, age, tumor location, size, pathological T, N status and differentiation. The results were similar to previous reports, even though the cell type and cutoff values for cfDNA were not the same [8,24,25]. Only the presence of lymphovascular invasion was associated with higher cfDNA levels in our study. Umetani and colleagues discovered that lymphovascular invasion or LN metastasis was associated with the integrity of cfDNA in breast cancer, but not in ESCC [26]. 

In our study, tumor relapse was associated with higher levels of cfDNA (*p* = 0.018). Tomochika et al. and Banki et al. also reported similar results in most ESCC cases and even in adenocarcinoma of the esophagus [23,25]. Interestingly, we found that ESCC patients with a higher concentration of cfDNA had a shorter DFS (*p* = 0.013), which was different from the report of Tomochika and his coworkers [23]. The reason might be due to different grouping methods of cfDNA in patients. In other studies, the relationship between DFS and cfDNA has infrequently been shown in esophageal cancer previously. Also, in the present study, patients with positive pathological N status, at a late stage and with the presence of lymphovascular invasion had poorer DFS. These factors have been frequently discussed in the literature [13,27].

A meta-analysis conducted by Ocaña et al. reported that high levels of total cfDNA were associated with worse OS in patients with solid tumors [22]. However, no esophageal cancer study was included in that meta-analysis. Tomochika et al. showed the cfDNA levels were not associated with OS [23]. In contrast, our study showed that patients with a lower concentration of cfDNA had a better OS, but not significantly (*p* = 0.164); thus, the results might be influenced due to patients who died of non-cancer disease after a five-year follow-up in the group with lower cfDNA levels. 

Banki and her coworkers reported that the cfDNA level in patients with esophageal cancer was significantly higher and the cfDNA level returned to normal after complete resection of the tumor [25]. Agostini et al. reported that the longer fragments of cfDNA were decreased in some patients with rectal cancer and cfDNA could be the responder of pre-operative chemoradiotherapy [28]. However, there was no similar study that reported the change of cfDNA after neoadjuvant radiochemotherapy in patients with esophageal cancer. Further study is needed to understand the role of cfDNA as the predictor for preoperative treatment and may identify patients who would benefit from the following surgery.

## 4. Materials and Methods 

### 4.1. Human Subjects and Clinical Data

The protocol of the present study was approved by the Institutional Review Board of Taipei Veterans General Hospital (IRB-TPEVGH No.: 2015-11-013BC, 30 November, 2015). Due to the retrospective nature of the study, requirements for patients’ informed consent were waived.

A total of 81 patients with ESCC who received esophagectomy with reconstruction at Taipei Veterans General Hospital between January 2006 and December 2013 were enrolled in this retrospective study. Those who had received preoperative chemoradiotherapy were excluded. Clinical data of all subjects were collected from patients’ medical charts, including age, gender, tumor location, pathological TNM stage, differentiation grade and related follow-up studies. Tumor staging was determined according to the 7th edition UICC/American Joint Committee on Cancer (AJCC) TNM staging system [29]. Follow-up studies, including biochemical measurement, CT scan of the chest and the brain, and whole body nuclear scan studies, were performed every three to six months within five years and then annually thereafter. Disease-free survival (DFS) was defined as the period from the date of surgery to the time of the first relapse (recurrence or metastasis). Overall survival (OS) was calculated from the date of surgery to the date of death because of any cause.

To confirm the ability of the cfDNA to discriminate the patients with esophageal cancer from healthy subjects, blood samples were obtained from the 95 healthy individuals without a history of cancer. These healthy subjects had no diagnosis of cancerous disease for at least two years after blood withdrawn.

### 4.2. Cell-Free DNA Isolation and Quantification of Circulating DNA Copy Number

All plasma samples for this study were obtained from the Biobank of Taipei Veterans General Hospital. Cell-free DNAs from plasma were extracted using the QIAamp DNA Tissue Kit and Minelute Virus Kit (Qiagen, Valencia, CA, USA) according to the manufacturer’s recommendations. The quality and quantity of DNA were measured by using the Nanodrop 1000 Spectrophotometer (Thermo Fisher Scientific, Waltham, MA, USA).

The quantity of cfDNA was represented by the copy number of the housekeeping gene cyclophilin (gCYC), a gene not known to be involved in cancer [30]. TaqMan qPCR assay (Thermo Fisher Scientific) was used to quantify the DNA copy number. Quantitative PCR was performed using TaKaRa Ex Master Mix. (Takara Bio, Shiga, Japan) Serially diluted standard DNAs were used to create a standard curve. Results were expressed as value of the threshold cycle (*C*_t_), i.e., the cycle number at which the PCR product reached the threshold of detection. The final *C*_t_ values were normalized based on the standard curve. The gCYC qPCR results were used to normalize plasma sample DNA to the number of DNA alleles per mL.

### 4.3. Statistical Analysis

A receiver operating characteristic (ROC) curve was drawn and the highest sensitivity and specificity were selected as the cutoff value. Relationships among categorical parameters were analyzed by Chi-square test or two-tailed Fisher’s exact test when the expected number in any cell was smaller than five cases. Kaplan–Meier survival curves were plotted and compared using the log-rank test. The impact of clinicopathologic features and circulating cfDNA concentration on DFS and OS were assessed using Cox regression multivariate analyses. Statistical significance was defined as *p* < 0.05. Statistical analyses were performed using SPSS 18.0 for Windows (SPSS, Inc., Chicago, IL, USA). 

## 5. Conclusions

The results of the present study demonstrated the cutoff value of cfDNA in ESCC patients with high sensitivity and specificity. Higher cfDNA levels in ESCC patients were associated with lymphovascular invasion, tumor relapse and shorter DFS. Circulating cfDNA could be a biomarker for predicting relapse of ESCC. Further evaluation of cfDNA is needed to determine its possible role in the mechanism responsible for metastasis or relapse of ESCC.

## Figures and Tables

**Table 1:** Comparison of clinocopathological parameters between patients with lower cell-free DNA (cfDNA) and higher cfDNA levels.

Variable		Lower cfDNA Levels (*n* = 40)	Higher cfDNA Levels (*n* = 41)	*p* Value
Age (years)				0.588
	<60	20 (50.0%)	23 (56.1%)	
	>60	20 (50.0%)	18 (43.9%)	
Gender				0.315
	male	33 (82.5%)	37 (90.2%)	
	female	7 (17.5%)	4 (9.8%)	
Smoking				0.860
	no	11 (27.5%)	12 (29.3%)	
	yes	29 (72.5%)	29 (70.7%)	
Alcohol drinking				0.218
	no	24 (60.0%)	19 (46.3%)	
	yes	16 (40.0%)	22 (53.7%)	
Betel nuts chewing				0.735
	no	32 (80.0%)	34 (82.9%)	
	yes	8 (20.0%)	7 (17.1%)	
Tumor location				0.312
	upper/middle	25 (62.5%)	21 (51.2%)	
	lower	15 (37.5%)	20 (48.8%)	
Tumor length				0.229
	<4 cm	21 (52.5%)	16 (39.0%)	
	≥4 cm	19 (47.5%)	25 (61.0%)	
Differentiation				0.460
	well	1 (2.5%)	3 (7.3%)	
	moderate	31 (77.5%)	31 (75.6%)	
	poorly	8 (20.0%)	7 (17.1%)	
Pathological T status				0.683
	T1–2	11 (27.5%)	13 (31.7%)	
	T3–4	29 (72.5%)	28 (68.3%)	
Pathological N status				0.224
	N0	18 (45.0%)	13 (31.7%)	
	N1–3	22 (55.0%)	28 (68.3%)	
Pathological stage				0.099
	I–II	22 (55.0%)	15 (36.6%)	
	III	18 (45.0%)	26 (63.4%)	
Lymphovascular invasion				0.033
	no	27 (67.5%)	18 (43.9%)	
	yes	13 (32.5%)	23 (56.1%)	
Perineural invasion				0.582
	no	27 (67.5%)	30 (73.2%)	
	yes	13 (32.5%)	11 (26.8%)	
Post-operative treatment				0.326
	no	20 (50.0%)	16 (39.0%)	
	yes	20 (50.0%)	25 (61.0%)	

**Table 2:** Survival analysis of prognostic factors influencing disease-free survival and overall survival after esophagectomy.

Variable		Disease-Free Survival		Overall Survival	
		Five-Year Survival Rate (%)	*p* Value	Five-Year Survival Rate (%)	*p* Value
cfDNA			0.013		0.164
	low	48.9		43.4	
	high	21.1		24.3	
Age (year)			0.199		0.174
	<60	39.7		39.4	
	>60	29.5		27.1	
Gender			0.645		0.901
	male	33.6		33.3	
	female	40.4		36.4	
Smoking			0.437		0.783
	no	41.1		34.8	
	yes	31.8		33.9	
Alcohol drinking			0.814		0.297
	no	38.2		32.8	
	yes	32.2		35.2	
Betel nuts chewing			0.614		0.589
	no	36.3		34.3	
	yes	31.0		20.4	
Tumor location			0.228		0.362
	upper/middle	44.0		40.3	
	lower	23.2		25.5	
Tumor length			0.493		0.659
	< 4cm	37.1		38.2	
	≥ 4cm	32.4		30.0	
Differentiation			0.387		0.274
	well	0		50.0	
	moderate	39.3		36.5	
	poorly	21.4		20.0	
Pathological T status			0.394		0.831
	T1–2	40.2		35.4	
	T3–4	32.4		33.0	
Pathological N status			0.025		0.025
	N0	49.5		50.4	
	N1–3	26.4		22.9	
Pathological stage			0.002		0.014
	I–II	51.7		49.5	
	III	21.3		20.3	
Lymphovascular invasion			0.004		0.359
	no	47.6		39.2	
	yes	20.0		26.9	
Perineural invasion			0.890		0.201
	no	34.9		27.9	
	yes	34.4		48.4	
Post-operative treatment			0.523		0.111
	no	29.5		25.0	
	yes	38.1		40.8	
